# The Three Integrated Phases of Left Atrial Macrophysiology and Their Interactions

**DOI:** 10.3390/ijms150915146

**Published:** 2014-08-27

**Authors:** Raman Mehrzad, Mohammad Rajab, David H. Spodick

**Affiliations:** 1 Department of Medicine, Steward Carney Hospital, Tufts University School of Medicine, 2100 Dorchester Avenue, Boston, MA 02124, USA; 2 Department of Medicine, Division of Cardiology, Virginia Commonwealth University Medical Center, Richmond, VA 23298, USA; E-Mail: mrajab@mcvh-vcu.edu; 3 Department of Medicine, Division of Cardiology, St. Vincent Hospital, University of Massachusetts Medical School, Worcester, MA 02124, USA; E-Mail: david.spodick@stvincenthospital.com

**Keywords:** left atrium, macrophysiology, phases, integrated, interactions

## Abstract

Our understanding of the left atrium is growing, although there are many aspects that are still poorly understood. The left atrium size as an imaging biomarker has been consistently shown to be a powerful predictor of outcomes and of different cardiovascular disorders, such as, but not limited to, atrial fibrillation, congestive heart failure, mitral regurgitation and stroke. Left atrial function has been conventionally divided into three integrated phases: reservoir, conduit and booster-pump. The highly dynamic left atrium and its response to the stretch and secretion of atrial neuropeptides leaves the left atrium far from being a simple transport chamber. The aim of this review is to provide an understanding of the left atrial physiology and its relation to disorders within the heart.

## 1. Introduction—Compelling Physiology

**Figure 1:**
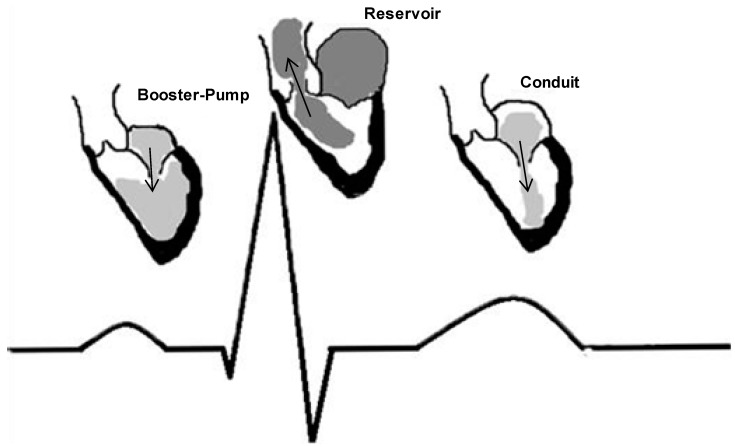
The three integrated phases of left atrium (LA) function in relation to the electrocardiogram (ECG).

The vastly dynamic left atrium (LA) and its response to the stretch and secretion of atrial neuropeptides leaves the LA far from being a simple transport chamber. Partial adjustment of fluid and hemodynamic balance is allowed by a balance of natriuresis, vasodilatation, and inhibition of the sympathetic and renin-angiotensin-aldosterone systems [1,2,3]. Furthermore, LA enlargement predicts stroke, transient ischemic attack (TIA), congestive heart failure (CHF), atrial fibrillation (AF) and other adverse cardiovascular outcomes [4,5,6]. Left atrial function has been typically divided into three integrated phases: reservoir, conduit and booster-pump (Figure 1) [7,8,9,10]. Reservoir: an expansion phase during left ventricular (LV) systole; the LA stores pulmonary venous return during LV contraction and isovolumic relaxation. Conduit: the LA transfers blood passively into the LV during ventricular diastole. Booster-pump: contractile component (when supraventricular rhythm is present); the LA actively contracts during the final phase of diastole and contributes between 15% and 30% of LV stroke volume [7,8,9,10]. Since the LA empties into the LV, size and function are very much dependent on the compliance of the LV, especially during diastole. In patients with ventricular dysfunction, the active contractile component of the LA has an important role to augment (“boost”) ejection force and stroke volume by increasing end-diastolic volume and pressures. In addition, in patients with reduced ventricular compliance, augmented left atrial booster function is one of the mechanisms compensating for decreased early filling. Diseases such as AF or in unsynchronized ventricular pacing, the loss of atrial contraction contributes to 20% reduction in cardiac output [11,12].

Increased reservoir function may play an important role in accelerating LV filling by helping to maintain an enhanced atrioventricular pressure gradient during diastole and also by increasing LA booster function through increased preload. This is supported by the LA reservoir and booster functions when augmented during exercise, whereas conduit function is not [13]. The conduit function of the LA increases when there is an isolated decrease in LA compliance. The ability to optimally redistribute LV filling among reservoir, conduit and booster-pump functions are a potentially important adaptation that may occur in the LA in response to changing hemodyanmics [14].

## 2. Adaptation

Left atrial preload is mainly volume dependent [13,15,16]; however, LA afterload is determined largely by its elastic properties and decreased LV pressure, as it increases with more severe LV diastolic dysfunction and elevated LV filling pressure [17]. When LA volume and pressure increase, there is an enlargement in LA size and concurrent gain in contractile shortening in the initial phase. Nevertheless, atrial fiber shortening and contractility begin to fail with progressive dilation of the LA, because it eventually leads to a threshold fiber length [18,19,20,21,22,23,24,25]. There are similar effects in the LV Frank-Starling curve, since beyond this threshold effect, further enlargement will only result in a decline of atrial function [26].

There is a dynamic and interactive relationship between LV and LA, evidenced by studies characterizing aging, athletic training, and multiple disease states [24,27,28,29]. *Aging*: In normal healthy subjects, LA volume index remains stable until the eighth decade despite the impaired LV diastolic relaxation that is apparent in the sixth decade, however, the latter is associated with a shift in phasic LA volumes, so that LA expansion index and passive emptying decrease while the active emptying volume increases [30]. *Athletes*: In contrast to aging, exercise increases the LA volume index, active and passive emptying in correlation with an improvement in LV diastolic properties, an increase in LV indexed mass and an increase in stroke volume. Thus, LA remodeling occurs in association with LV remodeling, and such LA enlargement represents the physiologic consequence of a global cardiac adaptation to the increased stroke volume associated with chronic and intensive exercise conditioning [31].

Lastly, the significant improvement in LA volume and function that is met after sinus rhythm is achieved with radiofrequency ablation for paroxysmal AF [32,33] or cardioversion of AF [34] is also evidence of the dynamic aspect of LA function and morphology.

## 3. Left Atrial Appendage: A Sometimes Deadly Attachment

Interestingly, the left atrial appendage (LAA) is embryologically and functionally distinct from the LA proper [35]. The LAA is derived from the LA and forms a blind pouch approximately 2–4 cm long. The neck of the LAA is relatively narrow and the endocardial surface is irregular due to pectinate muscles. It has been described as our most lethal human attachment [36]. It is closely related in its superior aspect to the pulmonary artery and inferomedially to the free wall of the left ventricle. As the left ventricle dilates during diastole, it fills the intrapericardial space, and it has been suggested that it may contribute to appendiceal emptying by compressing the inferomedial wall of the LAA between its free wall and the fixed pericardium [37]. In addition, ventricular filling creates an intracavitary suction effect, influencing emptying and filling of the atrium and consequently the appendage. The extent of emptying and filling of the LAA may be influenced more by changes in LV rather than LAA function [38]. This is a finding that could in part help to explain the higher incidence of AF-related stroke in patients with left ventricular dysfunction [39]. Moreover, in persistent AF, the strongest predictor of LAA thrombus is an increased left ventricular size [40].

Animal studies suggest that the LAA is more distensible than the LA proper [41]. In humans, clamping of the LAA during cardiac surgery results in an increase in LA pressure and dimension, as well as in transmitral and pulmonary diastolic flow velocities [42]. Because of its increased distensibility, the LAA may augment hemodynamic function by modulating LA pressure–volume relations in states of increased LA pressure and volume overload [43]. Atrial appendages contain about 30% of all the cardiac atrial natriuritic factor [44]. Both appendages can be selectively distended without altering the pressures in the right or left atrium or aorta, and distention causes an increase in heart rate (a reflex response with a vagal afferent path and a sympathetic efferent path) [45]. The presence of numerous myocardial stretch receptors in the LA, together with the fact that wall stress is amplified in the LAA due to its geometry, LAA becomes a more sensitive gauge of pressure changes than the LA proper [46].

The role of the LAA is most notable in AF and thromboembolic stroke [46]. Among those patients who sustained thromboembolic stroke, low flow velocities, spontaneous echo contrast, and thrombus have been identified in the LA appendage [31,47,48,49]. Atrial appendage flow velocities are often <50 cm/s in patients with AF, and this feature has been associated with thrombus formation and stroke [35]. As a strategy to lower thromboembolic events in AF patients, device closure of the LAA has been tested, and preliminary data suggest LAA occlusion with this system to be safe [50].

## 4. Frank-Starling Mechanism

Cardiac output (CO) increases in response to an increase in blood volume within the heart chambers. The increase in blood volume stretches the myocardial fibers, causing the cardiac muscle to contract more forcefully with increase in its mechanical performance. Intraatrial pressure controls atrial stretch. The Frank-Starling mechanism in the atrium is manifested in two phases. An increase in the amplitude of the calcium transient and a decrease in the time constant of Ca^2+^ transient decay are accompanied by the development of increased contraction force after stepwise increase of stretch. Thus, immediately after the stretch, the contraction force is increased, and an additional increase in force is followed instantly afterwards. An increase in muscle length results in a gradual augmentation of Ca^2+^ transient amplitude, which modulates the action potentials through increased Na^+^/Ca^2+^-exchange inward current. Troponin C is the calcium binding part of the contractile machinery, and is sensitive to muscle length. Experiments show that troponin C affinity change is needed to produce the immediate increase of contraction force and stretch, modulate the Ca^2+^ transient and stabilize the diastolic Ca^2+^ concentration [51]. The same mechanism that caused the normal physiologic responses to stretch could also generate arrythmogenic after potentials at high stretch levels in the model. Using a bivariate regression model with the assumption that variability is constant as a percentage of the expected value, a regression equation and graphs was developed that allowed calculation of a 95% prediction interval for several echocardiographic measurements as a function of the subject’s age and either body weight or body surface area [52].

With a stepwise decrease in the pacing heart rate, the LA dimension increases just before atrial contraction, and LA systolic shortening increases as well. Nevertheless, a decrease in the left ventricular filling volume during atrial systole is seen. As the pulmonary venous flow during atrial systole is directed toward the left atrium, the left atrial inflow volume from the pulmonary venous flow decreases, and the Frank-Starling mechanism operates with a decrease in the pacing rate. However, due to the decrease in pulmonary venous flow to the left ventricle via the left atrium, left ventricular filling decreases [53].

## 5. Left Atrial Distensibility

Left atrial relaxation, stiffness and contractility influence LA reservoir, conduit and contraction function. Furthermore, LA distensibility has prognostic significance in different cardiovascular diseases such as congestive heart failure (CHF), myocardial infarction and mitral regurgitation [54].

A study with 56 untreated patients in sinus rhythm, including 25 with previous myocardial infarction, 9 with hypertrophic cardiomyopathy, 11 with dilated cardiomyopathy, as well as 11 with chest pain syndrome as controls was done [55]. Peak first systolic velocity (PVS1), peak atrial systolic velocity (PVA), and their time-velocity integrals (PVS1-I and PVA-I, respectively) were calculated from the pulmonary venous flow velocity. Their analyses indicated that the peak first systolic velocity was closely related to percentage fractional LA relaxation, followed by mean pulmonary capillary wedge pressure. The peak first systolic velocity determined from the pulmonary venous flow velocity is closely related to parameters of LA relaxation, which may be determined by transesophageal echocardiography, and the ratio of peak atrial systolic velocity to peak first systolic velocity is useful for non-invasive evaluation of the LA pressure [55].

*In vivo*, regional differences in atrial distensibility may play an important role in modulating systolic and diastolic function of the LA. In the presence of LA pressure and/or volume overload, LAA becomes more compliant than the LA main chamber. Pericardiectomy increases LA compliance and early left ventricular filling rate and is accompanied by a relatively greater augmentation in conduit than reservoir function of the LA [56].

## 6. Pathological Changes

### 6.1. Left Atrial Function and Neuroendocrine Dependence

Control of heart rate, blood pressure, cardiac output, and regional blood flow are all affected by neurohormonal regulation [57]. In patients with CHF, plasma concentrations of brain natriuritic peptide, ANP, noradrenaline, renin and angiotensin-II are increased to compensate for the systemic failure caused by the heart. However, chronic activation of these hormones can cause negative cardiovascular effects such as LA remodeling and reduce atrial contractility with proarrhythmic and prothrombotic effects accordingly [58,59,60,61,62,63,64].

### 6.2. Atrial Fibrillation

Since synchronized atrial contraction is lost during AF, cardiac output will decline subsequently. To compensate for the decrease in stroke volume, the atrial pressure increases [65]. Moreover, after the introduction of AF, the diastolic atrial compliance decreases in both right and left atria [66]. Due to the hemodynamic load from the increase in atrial pressure, the size of the LA increases which in turn predisposes to AF [67]. Nevertheless, AF itself can result in atrial enlargement. Studies show that the LA is slightly dilated in patients with paroxysmal AF [68], and in patients with lone AF, a slow progressive enlargement of the LA size is usually noted independent of any changes in ventricular size or function [69]. As a consequence of left atrial enlargement, it may convey additional risk of stroke in patients with AF [70]. Thus, enlargement of the LA plays a role in AF, and therefore maintenance of sinus rhythm is important to prevent complications [71].

### 6.3. Importance of the Left Atrial Appendage

The LAA is a remnant of the original embryonic LA. Its interest has grown over the past years mostly due to the mechanical antithrombotic intervention strategies. The LAA has been found to be the source of >90% of thrombi in patients with nonvalvular AF [72]. Thus, prevention of thrombus formation by “eliminating” the LAA, through various methods is a novel therapeutic target for stroke prevention, especially in patients with contraindications to anticoagulation therapy [73]. However, LAA is important in regulating intravascular volume status and hemodynamic conditions such as mediating thirst, maintaining cardiac output, release of ANP and could not be a useless appendage [74].

### 6.4. Mitral Valve Disease

Left atrium function is influenced by changes in LA afterload. In patients with mitral stenosis (MS), the obstructed valve leads to a pressure gradient across the valve in diastole which causes an elevation in LA pressure and LA dilatation [11].

This early increase in LA pressure leads to LA enlargement, predisposing patients to AF and systemic embolism [75]. The increase in heart rate, as a result of new onset AF, leads to reduced CO. However, the atrial booster-pump function contributes less to ventricular filling in MS than in the normal heart, and the loss of atrial pump function is less important than the effect of increasing heart rate as the cause of decompensation during AF [76].

In acute mitral regurgitation (MR), the LA pressure increases quickly with atrial shortening and chamber dilatation as a result of the atrial muscle’s use of the Frank-Starling mechanism. The geometry of the mitral opening highly affects the degree of regurgitation, and a decrease in back-flow of blood with vasodilator therapy or with positive inotropic agents may be largely related to a decrease in the size of the left ventricle cavity. This brings components of the mitral apparatus closer together and increases its functional capability [77].

During chronic MR, a gradual increase in LA size is noted and this enables a more effective booster-pump function. However, LA pressure increases in the late phase of the disease, which leads to subsequent LA enlargement that predispose to AF, thromboembolism, pulmonary hypertension and right-sided heart failure as long term complication [14].

### 6.5. Hypertension

Hypertension is associated with left ventricular hypertrophy, myocardial fibrosis and alteration in calcium handling [78,79], which ultimately would lead to relaxation disorder and diastolic dysfunction [80,81]. As a consequence, blood flow from the LA is impaired which lead to LA enlargement. This phenomenon is well documented even in early hypertension [82]. Left ventricular mass and severity of diastolic dysfunction have been positively correlated to the size of LA [83,84,85]. Hypertension “accelerates” the normal aging process of the LA. Hypertensive patients as early as decade four, have similar LA size to that of normal controls in decade eight. This premature increase in LA volume increases the risk of AF development in hypertensive patients [4,83,86]. Hypertension also has been shown to decrease LA strain, a marker of LA reservoir function, and this is independent of LV hypertrophy. However, this decrease in reservoir function can be normalized with the use of renin-angiotensin system inhibitors if atrial enlargement has not yet occurred [87].

### 6.6. Myocardial Infarction

Left ventricular ejection fraction is usually decreased with myocardial infarction of the left ventricle. However, despite this decrease in ejection fraction, the stroke volume is usually maintained by an increase in left atrial booster function, resulting in remarkable rise in end-diastolic left ventricular volume. This inverse relationship in function between the LV and LA has been manifested by a significant increase in LA contractility during a decrease in LV peak systolic pressure and dp/dtmax [88]. The average atrial contribution to LV end-diastolic volume was 11.9% in normal subject compared to15.4% in patients with myocardial infarction; while the contribution to the LV stroke volume was 21.7% *vs.* 35.1%, respectively [89]. The above contribution could only be seen if the LA is not affected by the same ischemic event as it could happen during proximal left circumflex artery stenosis that render the LA ischemic and unable to augment its booster-pump function which exacerbates hemodynamic compromise [90]. However, with further LA enlargement and substantial increases in LA pressure, these compensatory mechanisms fail. Subsequently LA stroke volume increases causing augmented passive emptying [23,91] whilst contractile function is reduced [11]. Furthermore, increased LA volume index following an acute myocardial infarction was shown to be an independent predictor of mortality, independent of clinical variables and LV systolic and diastolic function [92].

### 6.7. Congestive Heart Failure

As mentioned above, LA reservoir and increased contractility counterbalance the initial impairment in LV filling. However, as the LV dysfunction progresses, these mechanisms become of limited effect and are replaced by the conduit function of the LA [23]. This could happen when the near-maximum left ventricular diameter is reached early in diastole with increased preload, rendering atrial contraction of little contribution to end diastolic ventricular volume [93].

In patients with idiopathic cardiomyopathy, compared to ischemic cardiomyopathy, atrial reservoir and contractile function were differentially reduced in the idiopathic cardiomyopathy group, highlighting the more generalized involvement of left atrium and ventricle in idiopathic cardiomyopathy [94].

Patients with hypertrophic cardiomyopathy have reduced atrial strain for all phases of atrial function, whilst atrial contractile function had an additive prognostic value in predicting the type of LV hypertrophy [95]. Additionally, strain derived atrial contractile function was the only independent predictor of heart failure symptoms (by the New York Heart Association class) in patients with hypertrophic cardiomyopathy [96].

### 6.8. Obstructive Sleep Apnea

Obstructive sleep apnea (OSA) contributes to the deterioration of LV diastolic function that can lead to atrial myocardial overstretching and enlargement, which could be associated with increased cardiovascular risk [97]. OSA causes a significant increase in LA volume that correlates with the severity of the disease, the more obstruction the larger LA volume. In addition, OSA causes a reduction in average and lateral mitral annular early diastolic velocities with an increase in average and lateral late diastolic velocities [98].

## 7. Conclusions

LA function is not fully investigated and in some aspects poorly understood. However, it has a powerful role in the cardiovascular system and in various heart disorders, and a growing understanding is crucial. Our review attempts to investigate the physiology of the LA and its associations in disease states. Consequently, in disease conditions, the LA augments failing left ventricular filling, and the LA may undergo failure itself. On this basis, LA function should be assessed since this might contribute to the evaluation of the current disease state, severity and outcome. Through different techniques, LA function can be evaluated in normal subjects, and in patients at rest or after pharmacological interventions. By measuring LA dimension and pressure one can evaluate LA function. An increase in the LA dimension and pressure and shift of the LA pressure-dimension curve with a parallel decrease in LA stroke work indicates LA dysfunction. After pharmacological interventions, changes in LV filling are attributed not only to LV diastolic function improvement but also to coordinated changes in LA function. The compensatory response to decreased early filling is increased atrial kick. An increased load on the LA, which may cause intrinsic LA dysfunction, can result in loss of kick. Left atrium dysfunction decreases LV filling in patients with LV dysfunction.

## Abbreviations

LA, Left atrium; CO, Cardiac output; CHF, Congestive heart failure; AF, Atrial fibrillation; LV, Left ventricle; MS, Mitral stenosis; MR, Mitral regurgitation; and OSA, Obstructive sleep apnea.
